# 

**Published:** 1916-08

**Authors:** 


### CONTENTS.

ORIGINAL COMMUNICATIONS—

Infantile Spinal Paralysis, (Anterior Poliomyelitis)—by Theodore Toepel, M. D.__ 197

The Treatment of Infectious Diarrheas in Infancy—by W. W. Harper, M. D., Selma, Ala.____ 202

Laboratory Findings as an Aid to Diagnoses. —by Miss Nannie Homilton, Birmingham, Ala.____ 206

EDITORIAL ____ 215

SELECTIONS AND ABSTRACTS____ 206

